# Monitoring COVID-19 in Belgian general practice: A tool for syndromic surveillance based on electronic health records

**DOI:** 10.1080/13814788.2023.2293699

**Published:** 2024-01-08

**Authors:** Bénédicte Vos, Laura Debouverie, Kris Doggen, Nicolas Delvaux, Bert Aertgeerts, Robrecht De Schreye, Bert Vaes

**Affiliations:** a Health Services Research, Sciensano, Brussels, Belgium; b Department of Public Health and Primary Care, KU Leuven, Leuven, Belgium

**Keywords:** Syndromic surveillance, covid-19, general practice, influenza-like illness, acute respiratory infection

## Abstract

**Background:**

COVID-19 may initially manifest as flu-like symptoms. As such, general practitioners (GPs) will likely to play an important role in monitoring the pandemic through syndromic surveillance.

**Objectives:**

To present a COVID-19 syndromic surveillance tool in Belgian general practices.

**Methods:**

We performed a nationwide observational prospective study in Belgian general practices. The surveillance tool extracted the daily entries of diagnostic codes for COVID-19 and associated conditions (suspected or confirmed COVID-19, acute respiratory infection and influenza-like illness) from electronic medical records. We calculated the 7-day rolling average for these diagnoses and compared them with data from two other Belgian population-based sources (laboratory-confirmed new COVID-19 cases and hospital admissions for COVID-19), using time series analysis. We also collected data from users and stakeholders about the syndromic surveillance tool and performed a thematic analysis.

**Results:**

4773 out of 11,935 practising GPs in Belgium participated in the study. The curve of contacts for suspected COVID-19 followed a similar trend compared with the curves of the official data sources: laboratory-confirmed COVID-19 cases and hospital admissions but with a 10-day delay for the latter. Data were quickly available and useful for decision making, but some technical and methodological components can be improved, such as a greater standardisation between EMR software developers.

**Conclusion:**

The syndromic surveillance tool for COVID-19 in primary care provides rapidly available data useful in all phases of the COVID-19 pandemic to support data-driven decision-making. Potential enhancements were identified for a prospective surveillance tool.

## KEY MESSAGES

Data extracted daily from electronic medical records can be used to monitor the COVID-19 pandemic in general practice.

The Barometer provided rapidly available data to support data-driven decision-making.

Improvements such as a greater standardisation were identified for a potential future tool using the same technology.

## Introduction

When COVID-19 hit, impacting the life and health of millions of people, general practitioners (GPs) were more than ever the first point of contact for health problems [1]. During the pandemic, GPs’ primary role was to manage patients with symptoms of influenza-like illness (ILI) and acute respiratory infections (ARI), to distinguish COVID-19 from other possible infections (influenza, ARI) and to follow up with ill patients and refer them to hospital if necessary. Because at the beginning of the pandemic early symptomatic COVID-19 often included fever and cough at the beginning of the pandemic, ILI and ARI were used as early markers to monitor COVID-19 in primary care settings [2,3]. Therefore, GPs played an important role in monitoring the pandemic through syndromic surveillance to support data-driven decision-making [4].

During the COVID-19 pandemic, monitoring the public health was essential to anticipate spread and evolution, including hospital capacity surges [5]. Syndromic surveillance is a real-time tool for early identification and monitoring of infectious disease outbreaks in the community, tracking disease evolution and informing governments and policymakers on public health [4,6]. It can also provide reassurance that no outbreak has occurred [6]. To monitor the incidence of acute syndromes, data using standardised clinical terminology from general practices are preferred [7].

Belgian primary care settings have a history of surveillance [8,9]. During the first wave of the COVID-19 pandemic (March 2020-Sept. 2020), GPs were asked to count and record the daily number of patient consultations and the percentage related to respiratory problems. This first COVID-19 surveillance tool helped monitor the pandemic in Belgian general practices [10].

In September 2020, we developed an updated COVID-19 syndromic surveillance tool, called Barometer, based on the aggregation and centralization of data from electronic medical records (EMR) in Belgian general practices, to monitor the pandemic. This paper’s objective was to present our experience with a COVID-19 syndromic surveillance tool (Barometer) in Belgian general practices. This study will highlight the lessons learned from a surveillance tool set up during a health crisis for future epidemics.

## Methods

### Design and setting

We implemented a nationwide observational prospective study in Belgian general practices. Belgium is a federal state comprising three regions: Flanders in the north, Wallonia in the south and the Brussels-Capital Region in the country’s centre. In 2021, Belgium had 11,935 practicing GPs [11].

### Recruitment

All Belgian general practices with electronic medical record (EMR) software were eligible to participate in the study. We recruited voluntary GPs from September to October 2020 in different ways: invitations were sent to GPs who participated in the initial version of the COVID-19 surveillance tool, as well as professional association newsletters and promotional advertisements in specialised journals. A financial incentive was provided through the National Institute for Health and Disability Insurance (NIHDI) from 26/10/2020 to 31/03/2021 and GPs were paid based on their participation level: sending COVID-19 related data *via* the Barometer 4-5 days a week was considered high participation, and 2-3 days a week was considered medium participation. The results presented in this paper relate to the incentive period.

### Data collection and analysis

We collected aggregated data at the general practice level using electronic Forms (eForms) providing a uniform and secure electronic exchange of structured data between healthcare professionals and healthcare organisations. The statistical module within the EMR was used to run standardised queries and extract data from the EMR of each practice and compile it into the eForm, which was then transferred to a secured and centralised platform: Healthdata [12]. To participate, GPs ran an audit in their EMR system at the end of the day (or by 10am the next day), which generated the number of recorded diagnostic codes related to COVID-19 for each physical or phone contact. These numbers were manually copied to the eForm by the GP. We expected one eForm per day per practice (also in practices with more than one GP) and if more were sent, only the last one was kept. Safety and privacy were respected, as GPs sent aggregated data to the centralised platform [13].

We collected two types of data: epidemiological data and practice identification data. Epidemiological data were based on recorded diagnostic codes in the EMR system (using ICPC-2 and ICD-10 codes as the standard coding system): suspected COVID-19, confirmed COVID-19, ARI and ILI (Table 1) [14]. As coding is not mandatory, the daily percentage of coded diagnoses was also collected to assess the coding behaviour of the general practice. Practice identification data included postal and email addresses and the GP’s unique professional number (NIHDI number). To assess the patient population coverage by participating practices, the number of active patients who had at least one contact with the practice in the two years prior to the study was collected. These two years was decided by consensus with scientific experts in the field (GPs, public health experts and NIHDI representatives). We performed the analyses on the eForms that met the consistency criteria we established (Table 2). We extrapolated epidemiological data, i.e. number of contacts for suspected COVID-19, confirmed COVID-19, ARI and ILI, to the general population based on the number of active patients per practice and we presented epidemiological indicators per 100,000 inhabitants. Graphs show the 7-day rolling average. Because no data were provided on weekend days, we interpolated raw data for Saturdays (with the day before) and Sundays (with the day after); for holidays during the week (Monday to Friday), we interpolated with the average of the days before and after.

**Table 1.** Epidemiological data – diagnostics and codes.

Diagnostics	Codes
Suspected COVID-19	ICPC-2 R80 ICD-10 J11.1
Confirmed COVID-19	ICPC-2 A77 ICD-10 B34.2
ARI	ICPC-2 H71, ICPC-2 R74, ICPC-2 R75, ICPC-2 R76, ICPC-2 R77, ICPC-2 R78, ICPC-2 R81
ILI	ICPC-2 R80

ARI: acute respiratory infection; ICD: international classification of diseases; ICPC: international classification of primary care; ILI: influenza-like illness.

**Table 2.** Criteria to include electronic eForms in the study.

Validation criteria
Number of ARI, ILI, or COVID-19 (suspected/confirmed) cases [each] < number of active patients Percentage of coded diagnoses ≥ 70% and ≤ 100% Number of suspected COVID-19 cases ≤ number of influenza syndromes Daily rates of ARI, ILI, confirmed COVID-19 cases [each] ≤ 2000/100,000 inhabitants

ARI: acute respiratory infection; ILI: influenza-like illness.

We interpreted results together with data from two other Belgian population-based sources, commissioned by the Belgian government: new laboratory-confirmed COVID-19 cases and hospital admissions for COVID-19. All confirmed COVID-19 cases were based on PCR and antigen testing [15]. Hospital admissions due to COVID-19 were recorded daily through the Surge Capacity survey (patients with a positive COVID-19 test and admitted due to COVID-19) [16].

We used descriptive statistics to present the variables’ range and characteristics. Dickey-Fuller Unit Root Tests was used to verify non-stationarity of time series. After conducting the Johansen cointegration test, the Vector Error Correction Model (VECM) was used to compare trends and examine relationships between each variable from the official Belgian data and all variables collected by the Barometer. The estimation method used was Maximum Likelihood Estimation with significance level set at 5%. We used SAS 9.4 to perform statistical analyses.

### Qualitative analysis

Throughout the project, we also collected information on the process of the Barometer setup. We used several data sources: i) reports of meetings with members of GP associations, GPs, government representatives, software developers and academic partners, ii) feedback by email and phone call from participating GPs and iii) observations and experiences of scientific investigators of the project. We performed a thematic analysis and structured the results according to the emerging themes (technical aspects, project management, participation). In each theme, we presented positive components about the tool and possible areas for improvement.

## Results

### Targeted population and participation

Of 11,935 practising GPs in Belgium, 4773 participated to the project at least once (3469 in Flanders, 973 in Wallonia and 331 in Brussels), representing 2068 practices (1411 in Flanders, 516 in Wallonia and 141 in Brussels). More than 1000 practices participated nationally on 104 out of 110 workdays (94.5%). Participation rate was higher in Flanders than in the other regions, leading to a higher percentage of the arrondissement population (*n* = 43 arrondissements in Belgium) covered in Flanders: up to 65-70% in Flanders, up to 40% in Wallonia and around 30% in Brussels (Suppl. material).

### Results from the Barometer (contacts for ARI, ILI or suspected/confirmed COVID-19) and comparison with official COVID-19 data sources

**Figure 1.**
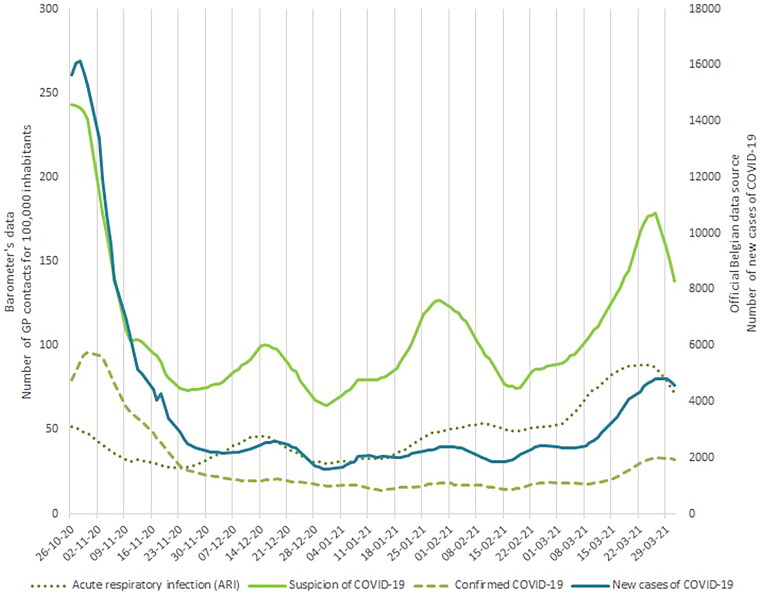
Comparison between the Barometer’s data on the number of contacts with a GP for 100,000 inhabitants for suspected or confirmed COVID-19 or acute respiratory infection (ARI) and the official Belgian data on new cases of COVID-19.

Table 3 presents the mean (SD) number of daily GP contacts per 100,000 inhabitants for each Barometer variable: 107 (± 40) for suspected COVID-19, 47 (± 17) for ARI and 28 (± 21) for confirmed COVID-19. The left scale in Figure 1 shows the number of daily GP contacts per 100,000 inhabitants for suspected/confirmed COVID-19 and ARI. We observed between 64 and 243 daily contacts per 100,000 inhabitants suspected COVID-19 during the study period. Data collection started at the peak of one wave and then three more minor (but growing) waves were observed in mid-December, late January and late March. The curve for ARI contacts followed a similar pattern, except for a less pronounced second wave. Daily contacts for confirmed COVID-19 were also highest at the beginning of the study (97 daily cases per 100,000 inhabitants) and then reached a plateau, rising slowly in March. The ILI curve is not shown in the figures because it generally followed the same curve for suspected COVID-19 (overlapping ICPC-2 codes).

**Table 3.** Description of the variables from the Barometer and the Belgian official data sources.

	Variables	*N*	Mean (SD)	Min-Max
Barometer	ARI	157	47,33 (17,26)	27,08–88,61
	Suspected COVID-19	157	106,84 (39,66)	64,41–242,91
	Confirmed COVID-19	157	28,18 (20,92)	14,18–97,23
Official sources	New cases of COVID-19	157	3588,89 (3156,92)	1590,29–16157,14
	Hospital admissions COVID-19	157	232,67 (160,42)	118,29–713,00

SD: Standard deviation.

**Figure 2.**
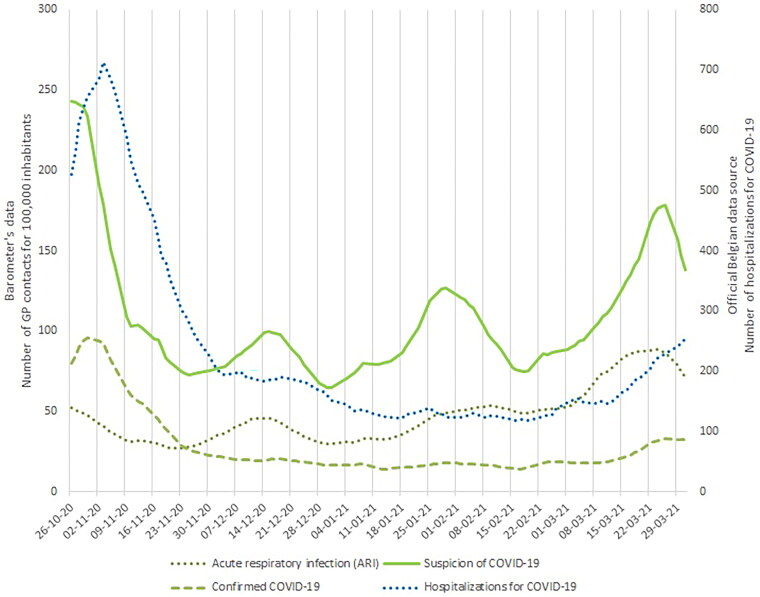
Comparison between the Barometer’s data on the number of contacts with a GP for 100,000 inhabitants for suspected or confirmed COVID-19 or acute respiratory infection (ARI) and the official Belgian data on hospitalisations for COVID-19.

We visually compared time series of the Barometer data with two other data sources: new laboratory-confirmed COVID-19 (Figure 1) and COVID-19 hospital admission (Figure 2). We observed that the number of contacts for suspected COVID-19 peaked at the same time in October and then followed a similar trend to the new laboratory-confirmed cases curves (Figure 1), with more pronounced bumps. The ARI curve followed a similar pattern to the curve of new cases (except for the first peak in the fall). The number of hospital admissions due to COVID-19 peaked in Belgium at the start of our data collection (Figure 2). This peak was about 10 days later than the peak of GP-patient contacts for suspected COVID-19. Time series analysis using VCEM suggests a significant unidirectional relationship between the two variables hospital admissions for COVID-19 and the Barometer’s confirmed COVID-19 cases (*p* < 0.0001) (Suppl. material). The test did not confirm that the Barometer variables predict the official laboratory-confirmed COVID-19 cases. However, a relationship exists in the opposite direction between the Barometer’s suspected COVID-19 and the hospital admissions, and the Barometer’s confirmed COVID-19 cases and the official laboratory-confirmed cases (Suppl. material).

### Positive and improving components of the Barometer

Table 4 summarises the main positive elements, such as the use of data for decision-making, linked to the data being quickly available (daily data collection with results published within a week of data entry) and based on broad participation. Table 4 also reports on some technical and methodological points to be improved, such as a greater standardisation between the different EMR software developers.

**Table 4.** Positive components and areas of improvement.

Positive points	Areas of improvement
*Technical details*	
Positive feedback from users of the eForm-based computer syndromic surveillance tool eForms: easy and quick to implement Rapidly available data (day-to-day data collection)	Dependence on EMR software company’s schedule ^a^Validation of technical developments to ensure standardisation among EMR software companies ^a^Full automation of data transfer between audit and eForm (to avoid manual transcription and mistyped data) ^a^Confirmation of reception of the forms by the Healthdata platform ^a^Unique identifier for practices (not only GPs)
*Project acceptability and participation*	
High level of participation maintained during the study period Voluntary participation (*note: impact of financial incentive unassessed*)	Support motivation (especially for long-term data collection)
*Data and data quality*	
Tool effective in identifying health changes (good match with official data) Visualisation of practice outcomes/interest in feedback tool	Strengthen training in the use of diagnostic codes in patients EMR Limiting inter-personal variability in coding ^a^Standardised calculation of the percentage of coded diagnoses (among software companies) External validity of data/comparability between different data sources
*Project management*	
Set of communication tools (daily email reminders to participating practices to send data, website/FAQ, tutorials) Collaboration with GPs or GPs associations (meetings, advice and discussions to improve the project)	Financial aspects: integration with other funding sources/systems or based on a representative sample of GP practices should be assessed Implementation of a more formal, multidisciplinary scientific monitoring committee from the early stages of the project

^a^ Should be fully implemented in a future project.

## Discussion

### Main findings

Our objective was to present the COVID-19 syndromic surveillance tool used in Belgian general practices to monitor the pandemic. We compared the results of the Barometer with official Belgian COVID-19 data and presented positive and improving components of this tool. Visual comparison of the Barometer’s ‘suspected COVID-19 results’ and the official laboratory-confirmed COVID-19 cases showed that both curves followed the same trend and rolling averages peaked at the same time. Therefore, we can say that the Barometer detected crisis fluctuations and outbreaks: the ‘suspected COVID-19 indicator’ is sufficiently sensitive. A similar conclusion can be drawn for the Barometer’s ‘ILI indicator’, as the two curves (ILI and suspected COVID-19) largely overlap (overlapping ICPC-2 codes) [14]. However, except for the peak at the start of the study, the Barometer’s ‘confirmed COVID-19 curve’ did not show the same fluctuations as the official new cases curve. We hypothesised that positive test results were not systematically coded in the EMR by the GP, due to the administrative burden, and therefore were not exhaustively included. The Barometer’s ‘ARI curve’ showed a similar trend to the curve for official new COVID-19 cases, except for the first peak (autumn 2020). This is explained by the similarities in symptomatology between ARI and COVID-19. The ‘ARI curve’ anticipates official new cases by about five days, which makes it an interesting indicator that can be intuitively used. The fewer contacts for ARI than for suspected COVID-19 and the absence of peak in the autumn of 2020 may be due to health restrictions (social distancing, mask-wearing, reinforced hand washing, etc.) [17].

### Methodological reflections

When visually comparing the Barometer data with national hospital admissions data, we perceived the Barometer’s ‘suspected COVID-19 curve’ peaking 10 days before hospital admissions (at the start of the study period). COVID-19 care may explain the differences between the two curves and particularly the absence of peaks in hospital admissions in the second part of the study period due to experience, improved knowledge of the disease and vaccination (which started in nursing homes in early 2021), patients became less likely to be hospitalised and follow up at home was instituted [18]. The Barometer’s ‘confirmed COVID-19 cases’ curve followed the same trend as the hospital admissions curve. Therefore, the Barometer anticipated the capacity surge in hospitals. For this reason, it was used in Belgian prediction models for hospitalisation and ICU admission [19]. The statistical analyses confirmed a relationship between the hospital admissions and the Barometer’s ‘confirmed COVID-19’ cases, supporting our statement.

Our study showed the relevance and feasibility of using extraction from EMR in general practices to monitor an epidemic, as previously demonstrated in another context [20]. However, due to methodological intricacies, the evolution of the curves is more relevant than the absolute incidence numbers. We used 7-day rolling averages to flatten ‘Monday peaks’ in our data due to the high number of contacts with GPs after weekends (a recognised situation in general practices) and to facilitate comparison with other data sources using this technique [21]. However, using 7-day rolling averages shifted the peaks of the curves slightly to the right [22].

A visual interpretation of the results when comparing time series allowed us to understand the behaviour and trends between the observed variables. However, these observations were not always supported by the results of statistical analysis. This may be due to the sample size or more complex relationships between the variables collected, e.g. predicting COVID-19 pandemic trends using a composite of several Barometer variables as predictors. Further investigation of the relationships between variables may inform surveillance systems and be incorporated into a forecasting model.

### Strengths and limitations

Several elements contributed to the high quality of our data. First, GP participation was high and stable throughout the study and the data covered a large part of the Belgian population. The willingness to be involved in the health crisis and the financial incentive may have contributed to this high participation level. Second, the national COVID-19 testing strategy remained stable throughout the study and did not affect our data. Third, using eForms and routine health data extracted from GPs’ software contributed to an easy-to-setup monitoring tool with rapid data availability. Some limitations and potential improvements have also been identified. First, manual entry of daily results by the GP in the eForms introduces a risk of errors. This can be solved by automated data extraction or pre-populated eForms. Second, the project’s progress depended on the time schedule of EMR software developers. Involving EMR software developers in the early stages of the project is therefore critical. Third, the diversity of EMR systems can affect the uniformity of collected data; therefore, we recommend implementing a standardised validation. Fourth, not all GPs are equally familiar with diagnostic codes. This can be improved through training and official guidelines recommendations.

## Conclusion

This study showed the feasibility of rapidly set up a real-time syndromic surveillance tool. A tool that uses routine data extraction and eForms provides quickly available data from primary care that are useful in all phases of the COVID-19 pandemic to support decision-making. The overall feedback on the use of the monitoring tool was positive; at the same time, several possible technical and methodological improvements were identified.

## Supplementary Material

Supplemental Material

## References

[1] Van den Bulck S, Crèvecoeur J, Aertgeerts B, et al. The impact of the covid-19 pandemic on the incidence of diseases and the provision of primary care: a registry-based study. Plos One. 2022;17(7):1. doi: 10.1371/journal.pone.0271049. PMC9258821 35793324

[2] Boëlle PY, Souty C, Launay T, et al. Excess cases of influenza-like illnesses synchronous with coronavirus disease (COVID-19) epidemic, France, march 2020. Euro Surveill. 2020;25(14)pii=:2000326. 32290901 10.2807/1560-7917.ES.2020.25.14.2000326 PMC7160441

[3] Du Z, Javan E, Nugent C, et al. Using the COVID-19 to influenza ratio to estimate early pandemic spread in Wuhan, China and Seattle, US. EClinicalMedicine. 2020;26:100479. doi: 10.1016/j.eclinm.2020.100479. 32838239 PMC7422814

[4] Kearon J, Risdon C. The role of primary care in a pandemic: reflections during the COVID-19 pandemic in Canada. J Prim Care Community Health. 2020;11:2150132720962871. doi: 10.1177/2150132720962871. 32985333 PMC7536478

[5] Bagaria J, Jansen T, Marques DF, et al. Rapidly adapting primary care sentinel surveillance across seven countries in Europe for COVID-19 in the first half of 2020: strengths, challenges, and lessons learned. Euro Surveill. 2022;27(26):2100864. 35775429 10.2807/1560-7917.ES.2022.27.26.2100864 PMC9248262

[6] Henning KJ. What is syndromic surveillance? MMWR Suppl. 2004;53:5–8. 15714620

[7] Jones NF, Marshall R. Evaluation of an electronic general-practitioner-based syndromic surveillance system–Auckland, New Zealand, 2000-2001. MMWR Suppl. 2004;53:173–178. 15714648

[8] Truyers C, Goderis G, Dewitte H, et al. The intego database: background, methods and basic results of a flemish general practice-based continuous morbidity registration project. BMC Med Inform Decis Mak. 2014;14(1):48. doi: 10.1186/1472-6947-14-48. 24906941 PMC4067630

[9] Miranda GHB, Baetens JM, Bossuyt N, et al. Real-time prediction of influenza outbreaks in Belgium. Epidemics. 2019;28:100341. doi: 10.1016/j.epidem.2019.04.001. 31047830

[10] Vaes B, Vos B, Foidart M, et al. Burden of COVID-19 on primary care in Belgium: a prospective nationwide observational study from march to august 2020. Arch Public Health. 2022;80(1):250. doi: 10.1186/s13690-022-01003-0. 36476628 PMC9730669

[11] IMA-AIM Intermutualistisch Agentschap – Agence Intermutualiste [Internet]. Brussels: squelette et graphisme: banlieues asbl; 2023. ATLAS AIM. [cited 2023 December 7]. Available from: https://www.ima-aim.be/IMA-Atlas.

[12] Healthdata.be Sciensano [Internet]. Brussels: sciensano; 2023. [cited 2023 December 7]. Available from: https://healthdata.sciensano.be/en/home.

[13] Delvaux N, Aertgeerts B, van Bussel JC, et al. Health data for research through a nationwide privacy-proof system in Belgium: design and implementation. JMIR Med Inform. 2018;6(4):e11428. doi: 10.2196/11428. 30455164 PMC6300317

[14] Verbeke M, Schrans D, Deroose S, et al. The international classification of primary care (ICPC-2): an essential tool in the EPR of the GP. Stud Health Technol Inform. 2006;124:809–814. 17108613

[15] COVID-19 Sciensano [Internet]. Brussels: sciensano; 2023. Covid-19 surveillance frequently asked questions page 11. [cited 2023 December 7].Available from: https://covid-19.sciensano.be/sites/default/files/Covid19/COVID-19_FAQ_ENG_final.pdf.

[16] Van Goethem N, Vilain A, Wyndham-Thomas C, et al. Rapid establishment of a national surveillance of COVID-19 hospitalizations in Belgium. Arch Public Health. 2020;78(1):121. doi: 10.1186/s13690-020-00505-z. 33292566 PMC7673251

[17] Huang QS, Wood T, Jelley L, et al. Impact of the COVID-19 nonpharmaceutical interventions on influenza and other respiratory viral infections in New Zealand. Nat Commun. 2021;12(1):1001. doi: 10.1038/s41467-021-21157-9. 33579926 PMC7881137

[18] Cornelis J, Van Grootven B, Irusta LA, et al. Remote monitoring of patients with COVID-19. Health services research (HSR). Brussels: Belgian Health Care Knowledge Centre (KCE); 2022. KCE Reports 354. https://kce.fgov.be/en/publications/all-reports/remote-monitoring-of-patients-with-covid-19

[19] Faes C, Hens N, Gilbert M. On the timing of interventions to preserve hospital capacity: lessons to be learned from the belgian SARS-CoV-2 pandemic in 2020. Arch Public Health. 2021;79(1):164. doi: 10.1186/s13690-021-00685-2. 34517923 PMC8436011

[20] Páscoa R, Rodrigues AP, Silva S, et al. Comparison between influenza coded primary care consultations and national influenza incidence obtained by the general practitioners sentinel network in Portugal from 2012 to 2017. PLOS One. 2018;13(2):e0192681. doi: 10.1371/journal.pone.0192681. 29438406 PMC5811043

[21] Post L, Culler K, Moss CB, et al. Surveillance of the second wave of COVID-19 in Europe: longitudinal trend analyses. JMIR Public Health Surveill. 2021;7(4):e25695. doi: 10.2196/25695. 33818391 PMC8080962

[22] Gallaway MS, Rigler J, Robinson S, et al. Trends in COVID-19 incidence After implementation of mitigation measures - Arizona, january 22-August 7, 2020. MMWR Morb Mortal Wkly Rep. 2020;69(40):1460–1463. doi: 10.15585/mmwr.mm6940e3. 33031366 PMC7561223

