# Supplementary material for: Monitoring COVID-19 in Belgian general practice: A tool for syndromic surveillance based on electronic health records
Source: Eur J Gen Pract. 2024 Jan 8;30(1):2293699. doi: 10.1080/13814788.2023.2293699 (PMC10776082; doi:10.1080/13814788.2023.2293699)
Supplement: Supplemental Material [file IGEN_A_2293699_SM2658.docx]

Supplemental material:

Figure: Percentage of population covered by participating practices (level: arrondissement)


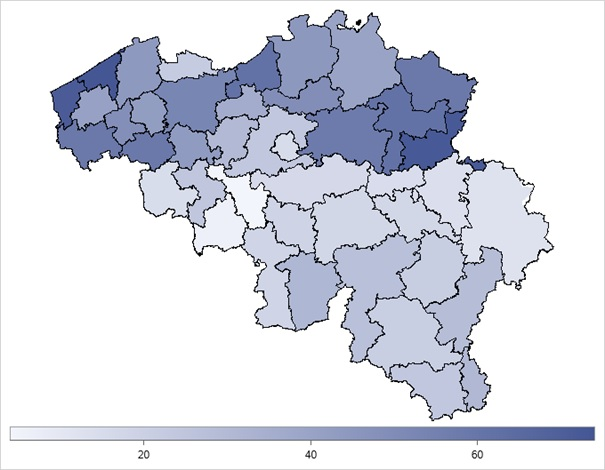


Vector Error Correction Model (VECM) Parameter Estimates

| Equation | Parameter | Estimate | Standard Error | t  Value | Pr > \|t\| | Variable |
| --- | --- | --- | --- | --- | --- | --- |
| D_New cases of COVID-19 | AR1_1_1 | -0.07151 | 0.02308 | -3.10 | 0.0023 | New cases of COVID-19(t-1) |
|  | AR1_1_2 | 137189 | 0.91598 | 1.50 | 0.1363 | ARI(t-1) |
|  | AR1_1_3 | 0.23255 | 0.70248 | 0.33 | 0.7411 | Suspicion of COVID-19 (t-1) |
|  | AR1_1_4 | 484338 | 330461 | 1.47 | 0.1449 | Confirmed COVID-19(t-1) |
| D_ARI | AR1_2_1 | 0.00008 | 0.00012 | 0.63 | 0.5274 | New cases of COVID-19(t-1) |
|  | AR1_2_2 | 0.00518 | 0.00477 | 1.09 | 0.2796 | ARI(t-1) |
|  | AR1_2_3 | -0.00222 | 0.00366 | -0.61 | 0.5441 | Suspicion of COVID-19 (t-1) |
|  | AR1_2_4 | -0.01223 | 0.01721 | -0.71 | 0.4785 | Confirmed COVID-19(t-1) |
| D_ Suspicion of COVID-19 | AR1_3_1 | 0.00030 | 0.00042 | 0.73 | 0.4688 | New cases of COVID-19(t-1) |
|  | AR1_3_2 | 0.04950 | 0.01653 | 2.99 | 0.0032 | ARI(t-1) |
|  | AR1_3_3 | -0.02703 | 0.01268 | -2.13 | 0.0346 | Suspicion of COVID-19 (t-1) |
|  | AR1_3_4 | -0.03487 | 0.05964 | -0.58 | 0.5596 | Confirmed COVID-19(t-1) |
| D_Confirmed COVID-19 | AR1_4_1 | 0.00040 | 0.00009 | 4.47 | <.0001 | New cases of COVID-19(t-1) |
|  | AR1_4_2 | 0.00489 | 0.00359 | 1.36 | 0.1752 | ARI(t-1) |
|  | AR1_4_3 | -0.00107 | 0.00275 | -0.39 | 0.6981 | Suspicion of COVID-19 (t-1) |
|  | AR1_4_4 | -0.05941 | 0.01294 | -4.59 | <.0001 | Confirmed COVID-19(t-1) |
| D_Hospitalizations for COVID-19 | AR1_1_1 | -0.11414 | 0.02134 | -5.35 | <.0001 | Hospitalizations for COVID-19(t-1) |
|  | AR1_1_2 | 0.03056 | 0.04046 | 0.76 | 0.4513 | ARI(t-1) |
|  | AR1_1_3 | -0.00195 | 0.03141 | -0.06 | 0.9507 | Suspicion of COVID-19 (t-1) |
|  | AR1_1_4 | 0.86021 | 0.18163 | 4.74 | <.0001 | Confirmed COVID-19(t-1) |
| D_ARI | AR1_2_1 | 0.00248 | 0.00247 | 1.01 | 0.3157 | Hospitalizations for COVID-19(t-1) |
|  | AR1_2_2 | 0.00323 | 0.00468 | 0.69 | 0.4908 | ARI(t-1) |
|  | AR1_2_3 | -0.00182 | 0.00363 | -0.50 | 0.6176 | Suspicion of COVID-19 (t-1) |
|  | AR1_2_4 | -0.01956 | 0.02099 | -0.93 | 0.3531 | Confirmed COVID-19(t-1) |
| D_ Suspicion of COVID-19 | AR1_3_1 | 0.02534 | 0.00829 | 3.06 | 0.0026 | Hospitalizations for COVID-19(t-1) |
|  | AR1_3_2 | 0.03967 | 0.01571 | 2.53 | 0.0126 | ARI(t-1) |
|  | AR1_3_3 | -0.02229 | 0.01220 | -1.83 | 0.0697 | Suspicion of COVID-19 (t-1) |
|  | AR1_3_4 | -0.19843 | 0.07053 | -2.81 | 0.0056 | Confirmed COVID-19(t-1) |
| D_Confirmed COVID-19 | AR1_4_1 | 0.00121 | 0.00205 | 0.59 | 0.5556 | Hospitalizations for COVID-19(t-1) |
|  | AR1_4_2 | 0.00230 | 0.00388 | 0.59 | 0.5548 | ARI(t-1) |
|  | AR1_4_3 | 0.00175 | 0.00301 | 0.58 | 0.5621 | Suspicion of COVID-19 (t-1) |
|  | AR1_4_4 | -0.02339 | 0.01741 | -1.34 | 0.1813 | Confirmed COVID-19(t-1) |
| *Prefix D_: differencing (non-stationary) variable as part of the process of fitting to the model*  *t value: t-statistic value*  *Pr(>\|t\|): p-value for t-test*  *ARI: acute respiratory infection* | | | | | | |
